# Indoleamine 2,3-Dioxygenase Activity in *Chlamydia muridarum* and *Chlamydia pneumoniae* Infected Mouse Lung Tissues

**DOI:** 10.3389/fcimb.2019.00192

**Published:** 2019-06-12

**Authors:** Dezső P. Virok, Tímea Raffai, Dávid Kókai, Dóra Paróczai, Anita Bogdanov, Gábor Veres, László Vécsei, Szilárd Poliska, László Tiszlavicz, Ferenc Somogyvári, Valéria Endrész, Katalin Burián

**Affiliations:** ^1^Department of Medical Microbiology and Immunobiology, Faculty of Medicine, University of Szeged, Szeged, Hungary; ^2^MTA-SZTE Neuroscience Research Group, Szeged, Hungary; ^3^Department of Neurology, Faculty of Medicine, Albert Szent-Györgyi Clinical Center, University of Szeged, Szeged, Hungary; ^4^Genomic Medicine and Bioinformatics Core Facility, Department of Biochemistry and Molecular Biology, Faculty of Medicine, University of Debrecen, Debrecen, Hungary; ^5^Department of Pathology, Faculty of Medicine, Albert Szent-Györgyi Clinical Center, University of Szeged, Szeged, Hungary

**Keywords:** IDO, iNOS, nitric oxide, *Chlamydia*, mouse, lung, interferon, interferon-inducible GTPases

## Abstract

*Chlamydia trachomatis* infections are the most prevalent sexually transmitted infections with potentially debilitating sequelae, such as infertility. Mouse models are generally used for vaccine development, to study the immune response and histopathology associated with *Chlamydia* infection. An important question regarding murine models is the *in vivo* identification of murine host genes responsible for the elimination of the murine and human *Chlamydia* strains. RNA sequencing of the *Chlamydia muridarum* infected BALB/c lung transcriptome revealed that several genes with direct antichlamydial functions were induced at the tissue level, including the already described and novel members of the murine interferon-inducible GTPase family, the CXCL chemokines *CXCL9, CXCL11*, immunoresponsive gene 1, nitric oxide synthase-2 (*iNOS*), and lipocalin-2. Indoleamine 2,3-dioxygenase 1-2 (*IDO1-2*) previously described potent antichlamydial host enzymes were also highly expressed in the infected murine lungs. This finding was novel, since *IDO* was considered as a unique human antichlamydial defense gene. Besides a lower level of epithelial cell positivity, immunohistochemistry showed that IDO1-2 proteins were expressed prominently in macrophages. Detection of the tryptophan degradation product kynurenine and the impact of IDO inhibition on *Chlamydia muridarum* growth proved that the IDO1-2 proteins were functionally active. IDO1-2 activity also increased in *Chlamydia muridarum* infected C57BL/6 lung tissues, indicating that this phenomenon is not mouse strain specific. Our study shows that the murine antichlamydial response includes a variety of highly up-regulated defense genes *in vivo*. Among these genes the antichlamydial effectors *IDO1-2* were identified. The potential impact of murine IDO1-2 expression on *Chlamydia* propagation needs further investigation.

## Introduction

*Chlamydiae* are obligate intracellular bacteria that propagate prominently in the epithelial cells of the respiratory and urogenital tract. The socioeconomic impact of *C. trachomatis* infection is significant. In developing countries, the ocular serovars of the species cause trachoma, the chronic infection/inflammation of the conjunctiva. Trachoma is the most important cause of preventable, infection-related blindness and in 2008 about 40 million people had active trachoma infection (Mariotti et al., 2009). Urogenital serotypes of *Chlamydia trachomatis* (*C. trachomatis*) are the most prevalent bacteria related to sexually transmitted infections (Centers for Disease Control Prevention, 2014), frequently leading to chronic infections with debilitating sequelae such as ectopic pregnancy and infertility. *C. trachomatis* infections can be treated effectively with macrolides and doxycycline (Kong et al., 2014), but the symptoms of urogenital infections are frequently mild and therefore the infection may be left untreated (Lallemand et al., 2016). Though the prevention of the infection with vaccination would be important, an effective vaccine has not yet been developed. Mouse models are the most frequently used ones for vaccine development, but the differences between the human and murine immune systems, including the so-called cell-autonomous immunity makes the mouse models difficult to compare with humans (Finethy and Coers, 2016). Cell autonomous immunity is an intrinsic feature of the host cells, which launches defense mechanisms that interfere with the growth of intracellular pathogens. Typically these defense genes are inducible, and interferon-gamma (IFNG) is a prominent inducer cytokine. It has been described previously that the major intracellular antichlamydial defense mechanism in human cells is the IFNG-induced IDO expression, which leads to the degradation of the intracellular tryptophan pool and eventually the death of the tryptophan-auxotroph *C. trachomatis* (Byrne et al., 1986). This elimination mechanism is effective *in vitro* for both the human *C. trachomatis* and the genetically closely related murine *Chlamydia* species *C. muridarum* (Roshick et al., 2006). Nevertheless *in vitro* data showed that IDO is not inducible by *Chlamydia* infection and/or IFNG in mouse epithelial cells (Roshick et al., 2006). Instead, microarray analysis of IFNG treated and *Chlamydia* infected murine epithelial cells revealed that the IFN-inducible GTPases are the suspected host genes that interfere with the developmental cycle of human *Chlamydia* strains (Nelson et al., 2005). Murine *Chlamydia* strain developed mechanism(s) to inactivate the GTPase response and render this elimination mechanism ineffective (Nelson et al., 2005; Coers et al., 2008). Despite this, the *C. muridarum* strain is rapidly eliminated from the murine cervicovaginal tract (Nelson et al., 2005), hence yet unknown elimination mechanisms exist in mice that are effective against the murine *Chlamydia* strain *in vivo*.

The aim of our experiments was to find murine defense genes that could be involved in the elimination of the murine *Chlamydia* strain. We chose a murine lung infection model, where the complexity of the *in vivo* environment—including the impact of a variety of cytokines and cell-cell interactions—could induce the expression of a diverse set of host genes. We performed an unbiased study, where we explored the inducible murine genes by screening the global gene expressions of the *C. muridarum* infected murine lungs.

## Methods

### Propagation of *Chlamydia pneumoniae* and *C. muridarum*

*Chlamydia pneumoniae* (*C. pneumoniae*) CWL029 strain from American Type Culture Collection (ATCC, Manassas, VA, USA) was propagated in HEp-2 cells (ATCC), as described earlier (Burián et al., 2003). *C. muridarum* strain Nigg (Nelson et al., 2005) was grown in McCoy cells (ECACC, London, UK). After partial purification and concentration the elementary bodies (EBs) were aliquoted in sucrose-phosphate-glutamic acid buffer (SPG) and stored at −80°C until use (Caldwell et al., 1981).

### Mice and Infection Conditions

Pathogen-free 6-week-old female BALB/c mice were obtained from the Charles River Laboratories (Hungary), C57BL/6 mice were obtained from BRC Animal House (Szeged, Hungary). The mice were maintained under standard husbandry conditions at the animal facility of the Department of Medical Microbiology and Immunobiology, University of Szeged, and were provided with food and water *ad libitum*. Before infection, the mice were mildly sedated with an intraperitoneal injection of 200 μl of sodium pentobarbital (7.5 mg/ml); they were then infected intranasally with 4 ×10^5^ IFU *C. pneumoniae* (BALB/c) or 1 ×10^3^ IFU of *C. muridarum* (BALB/c and C57BL/6) in 20 μl SPG buffer. Control mice were treated with 20 μl SPG buffer only. The mice were anesthetized and sacrificed 7 days after infection. The lungs were removed and homogenized with acid-purified sea sand (Sigma, St. Louis, MO, USA). Half of each homogenized lung was processed for total RNA extraction, and the other half was suspended in 1 ml of SPG for the detection of viable *Chlamydia* and to test the quantity of kynurenine and tryptophan. The lungs of three mice from both groups were fixed in 10% neutral buffered formalin solution (Sigma) for histopathological evaluation. The experiments were approved by the Animal Welfare Committee of the University of Szeged and conform to the Directive 2010/63/EU of the European Parliament.

### Culturing of *Chlamydia* From Mouse Lungs

Homogenized lungs from individual mice were centrifuged (10 min, 400 g), serial dilutions of the supernatants were inoculated onto McCoy cell monolayers and centrifuged (1 h, 800 g), and after a 48-h culture the cells were fixed with acetone and stained with monoclonal anti-*Chlamydia* LPS antibody (AbD Serotec, Oxford, UK) and FITC-labeled anti-mouse IgG (Sigma). The number of the recoverable *Chlamydia* inclusions was counted under a UV microscope and expressed as IFU/lung.

### *In vivo* IDO Inhibition by 1-Methyl-DL-Tryptophan

Seven days before infection with *C. muridarum* the drinking water of 8 weeks old female BALB/c mice (*n* = 4) was changed to that containing 2 mg/ml IDO inhibitor 1-methyl-DL-tryptophan (1-MT; Sigma), dissolved in 10 mmol/l NaOH supplemented with Stevia sweetener. Control mice (*n* = 4) received the Stevia-sweetened drinking water with 10 mmol/l NaOH without 1-MT. The solution was delivered in autoclaved water bottles, protected from light, and changed every other day. The infection of mice and the estimation of recoverable viable *C. muridarum* from the lungs at 7 days post infection were carried out as described previously.

### Total RNA Extraction and cDNA Synthesis

Total RNA was extracted from homogenized lung tissues of *C. muridarum* infected BALB/c (*n* = 3) and C57BL/6 mice (*n* = 5), *C. pneumoniae* infected BALB/c mice and uninfected controls with Tri Reagent according to the manufacturer's protocol (Sigma). Total RNA quantity (OD260) and purity (OD260/280) were measured by a NanoDrop spectrophotometer (Thermo Scientific, Waltham, MA, USA).

### cDNA Library Preparation and Sequencing

cDNA library for RNA-Seq was generated from 1 μg total BALB/c lung RNA using TruSeq RNA Sample Preparation Kit (Illumina, San Diego, CA, USA) according to the manufacturer's protocol. Single read 50 bp sequencing run was performed on Illumina HiScan SQ instrument (Illumina). CASAVA software was used for pass filtering and demultiplexing process. Sequenced reads were aligned to *Mus musculus* mm10 genome version using TopHat and Cufflinks algorithms and bam files were generated.

### Statistical and Functional Analysis of RNA-Sequencing Data

StrandNGS software (Agilent, Santa Clara, CA, USA) was used for the statistical analysis of RNA-sequencing (RNA-Seq) data. The aligned bam files were imported and DESeq algorithm was used in the quantification step to generate normalized gene expression data. Differentially expressed genes between *C. muridarum* infected lung samples (*n* = 3) and controls (*n* = 3) were determined using the Student's *t*-test combined with Benjamini-Hochberg FDR for multiple testing correction. Statistical significance was defined as *P*_Benjamini−Hochberg_ < 0.05. Library preparations, sequencing and data analysis were performed by UD-GenoMed Kft. and the Genomic Medicine and Bioinformatics Core Facility of University of Debrecen, Debrecen, Hungary. Large scale functional analysis of differentially expressed genes were performed by the Voronto software (Santamaría and Pierre, 2012). The Voronto method identifies the Gene Ontology terms and KEGG pathways that contain significantly enriched differentially expressed genes. Voronto software uses the so-called Voronoi tessellation to map ontology terms or pathways into a map-like structure. The cells of the map are the ontology terms, the closer terms being located closer to each other. Terms with a common ancestor are surrounded by a thicker line.

### Quantitative PCR Validation of the IDO1 and IDO2 RNA-Seq Data

For quantitative PCR (qPCR) 1 μg of total RNA was reverse transcribed using the Maxima Reverse Transcriptase according to the manufacturer's protocol with random hexamer priming (Thermo Fisher Scientific Inc. Waltham, MA, USA). qPCR was performed in a Bio-Rad CFX96 real-time system. The qPCR was performed with the SsoFast EvaGreen qPCR Supermix (Bio-Rad, Hercules, CA, USA) master mix and the murine specific primer pairs *IDO1*: 5′-GCTTCTTCCTCGTCTCTCTATTG-3′, 5′-TCTCCAGACTGGTAGCTATGT-3′; *IDO2*: 5′-CCTGGACTGCAGATTCCTAAAG-3′, 5′-CCAAGTTCCTGGATACCTCAAC-3′; *beta-actin*: 5′-TGGAATCCTGTGGCATCCATGAAAC-3′, 5′-TAAAACGCAGCTCAGTAACAGTCCG-3′. To check the amplification specificity, the qPCR was followed by a melting curve analysis. Threshold cycles (Ct) were calculated for *IDO1, IDO2* and *beta-actin* genes, and the normalized gene expressions were calculated by the ΔCt method (Ct_IDO1_-Ct_actin_ or Ct_IDO2_-Ct_actin_). Statistical comparison of qPCR data was performed by comparing the ΔCt values of uninfected and infected lung samples (*n* = 3) by using the Student's *t*-test as described earlier (Yuan et al., 2006).

### IDO1 and IDO2 Immunohistochemistry of *Chlamydia* Infected and Uninfected Mouse Lungs

IDO1 and IDO2 immunohistochemistry was performed on the BALB/c lungs that were used in the gene expression studies. Macroscopically inflamed lung sections and control lungs were cut and fixed in 10% formalin (Sigma). Fixed samples were cut into 4 μm sections. Tissue sections were first deparaffinized, followed by antigen retrieval and inhibition of endogen peroxidases using the EnVision FLEX Peroxidase-blocking reagent (Dako, Carpinteria, CA, USA). IDO immunohistochemistry was performed with a goat polyclonal anti-IDO1 antibody (Sigma) and a rabbit polyclonal anti-IDO2 antibody (Bioss, Woburn, MA, USA) followed by HRP-conjugated anti-goat rabbit (Dako) and anti-rabbit goat secondary antibodies (Dako), respectively.

### Detection of Tryptophan and Kynurenine Concentrations in Lung Tissues

Infected and control BALB/c and C57BL/6 mouse lung tissues were homogenized with sterile sand (Sigma), and dissolved in 700 μl 1 × PBS. The samples were sonicated 2 ×1 min, vortexed and centrifuged at 500 g for 5 min. Twenty microliters of internal standard (3-nitro L-tyrosine (3NLT, Sigma), 2 μM final concentration in the sample) in 2.5 w/w% perchloric acid was added to 480 μl supernatant and mixed with 500 μl perchloric acid (2.5 w/w%). The samples were subsequently centrifuged at 12,000 g for 10 min at 4°C, and the supernatants were collected for measurement. The tryptophan and kynurenine concentrations of the samples were quantified based on the work of Hervé et al. (1996) with slight modifications (Veres et al., 2015). In the high-performance liquid chromatography (HPLC) analysis, the peak area responses were plotted against the corresponding concentration, and the linear regression computations were carried out using the least squares method with the R software package (R Core Team, 2014).

## Results

### *Chlamydia* Infection and *Chlamydia*-Induced Histopathology

**Figure 1:**
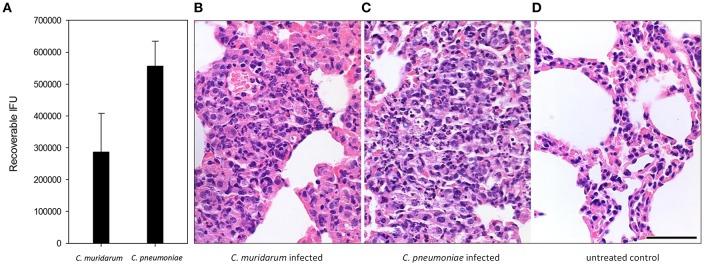
*Chlamydia* infection and *Chlamydia*-induced histopathology in BALB/c mouse lung tissues. Recoverable IFUs from *C. muridarum* infected and *C. pneumoniae* infected mouse lungs at 7 days post infection **(A)**. Haematoxylin-eosin staining of *C. muridarum* infected **(B)**, *C. pneumoniae* infected **(C)** and uninfected **(D)** lung tissues. Bar is 50 μm.

In our animal model different doses of the two *Chlamydia* strains were used (4 ×10^5^ IFU of *C. pneumoniae* and 1 ×10^3^ IFU of *C. muridarum)*, based on the results of our former experiments (Burián et al., 2003; Kis et al., 2008) where the lower dose of the murine pathogen *C. muridarum* induced similar growth and histopathology than the human pathogen *C. pneumoniae*. Indeed, recoverable IFUs from the *C. muridarum* and *C. pneumoniae* infected BALB/c lungs were similar at 7 days post infection (Figure 1A). Both infections induced a lymphoid hyperplasia, with the interstitial accumulation of lymphoid, plasmocytoid cells, and macrophages in the widened bronchus walls. The histology picture was consistent with the formation of inducible bronchus associated lymphatic tissue (iBALT). At this time of the infection, the presence of neutrophils were marginal, the major leukocyte populations were lymphocytes and macrophages (Figures 1B,C). Control lung tissue showed thin alveolar septa without the clear presence of inflammatory cells, but a small number of pulmonary macrophages could be detected (Figure 1D).

### Global Gene Expression Changes in the *C. muridarum* Infected Mouse Lung Tissues

To explore the global gene expression changes induced at the tissue level by the murine *Chlamydia* strain we performed an Illumina next generation RNA sequencing of BALB/c mouse lung tissues infected with *C. muridarum* 7 days post infection. RNA-seq analysis revealed that 755 murine genes had a higher expression and 251 genes had a lower expression than the uninfected control. The extent of up-regulation and the number of up-regulated genes was higher (1.48–345 fold), than in the case of the down-regulated genes (1.5–14.36 fold). The most highly up-regulated gene was the *CXCL11* (*I-TAC*), and several cytokines/ chemokines were among the highly upregulated genes including *CXCL9* (*MIG*), *CXCL10* (*IP-10*), *CCL8* (*MCP2*), *CCL2* (*MCP1*), *IFNG, IL21, IL10*, as well as already described defense genes *IRG1, IIGP* and *IDO1*. The most highly down-regulated gene was cDNA sequence *BC023719* with a 14.36 fold of down-regulation, and the functions of the most highly down-regulated genes were diverse. The list of differentially expressed genes can be found in the Supplementary Table 1.

**Figure 2:**
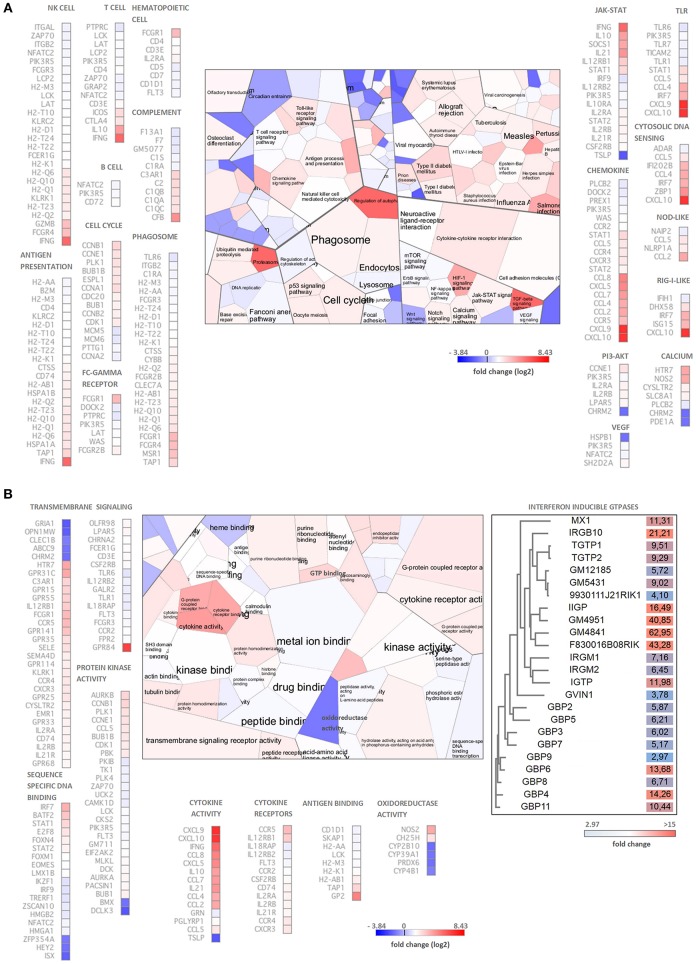
Functional analysis of the *C. muridarum* infection altered genes by the Voronto method. **(A)** Analysis of significantly enriched KEGG pathways containing differentially expressed genes. **(B)** Gene Ontology analysis of significantly enriched molecular function terms containing differentially expressed genes. The differentially expressed IFN-inducible GTPases are grouped based on their amino acid sequences shown separately with their fold of up-regulation. *Chlamydia* induced inflammation, immunity and antichlamydial defense related Gene Ontology terms and KEGG pathways and the corresponding genes are shown separately. Cell colors of the Voronto diagrams show the mean fold change (log2) of the differentially expressed genes related to the particular Voronto cells. Both for the Voronto cells and the genes, the log2 fold changes are color coded according to the scales. Note that the colors of the up-regulated genes ranging from light blue to red, while the down-regulated genes are dark blue.

Functional analysis of the differentially expressed genes using the Voronto method revealed several KEGG pathways that are related to *Chlamydia*-induced inflammation and antichlamydial innate and adaptive defense responses (Figure 2A). Several up-regulated genes rendered to cell-type specific KEGG pathways such as T-cell, B-cell, NK-cell and hematopoietic cells-specific pathways indicating the influx of these leukocytes into the infected lung tissues. Indeed, increased expressions of various cell-specific genes and CD markers were detected such as T-cell markers *CD3, CD4, CD5, CD6, CD7, CD226*, B cells markers *CD5, CD7*, dendritic cell marker *CD4*, NK-cell markers *CD4, CD7*, natural killer cell lectin-like receptors (*KLRA2, KLRB1F, KLRK1, KLRI2, KLRC2, KLRD1*) and macrophage-specific genes *CD4, MSR1*, and *MPEG1*, indicating the influx and/or local proliferation of these cells in the infected lung tissue. Interestingly, when the up-regulated genes were compared to the Mouse Gene Atlas gene expression database (Kuleshov et al., 2016), the most significant overlap was detected with the LPS treated macrophage gene expression (data not shown), indicating the active involvement of activated macrophages in the *Chlamydia*-induced gene expression changes. We detected several highly upregulated chemokines, that could induce the cellular influx, including the lymphocyte chemokines *CCL2* (*MCP1*), *CCL5* (*RANTES*), *CCL8* (*MCP2*), *CXCL9* (*MIG*), *CXCL10* (*IP10*), *CXCL11* (*I-TAC*), the monocyte chemokines *CCL2, CCL4* (*MIP1B*), *CCL7* (*MCP3*), *CCL8* (*MCP2*), *CXCL10* and the neutrophil granulocyte chemokine *CXCL5* (*ENA78*). Enhancing the effects of chemokines, various chemokine receptors were also up-regulated including the MCP1 receptor *CCR2*, the MCP1-MIP1-RANTES receptor *CCR4*, the MIP1B receptor *CCR5* and the MIG-IP10-I-TAC receptor *CXCR3*.

Gene Ontology molecular function analysis supported the KEGG pathway analysis (Figure 2B). The fact that various “cell cycle” pathway related genes were found to be up-regulated indicates the accelerated division of activated cells including the induction of BALT. The cellular activation is induced by ligand-receptor interactions in both adaptive and innate immunity. Indeed, among the largest functional groups identified were “cytokine activity,” “transmembrane signaling” and “protein kinase activity” containing prominently up-regulated and highly up-regulated cytokines, cytokine receptors and downstream signaling genes. Several adaptive immunity related genes, mainly MHC-I antigen presentation related genes were up-regulated. The pathogen recognition/proinflammation pathways such as the toll-like receptor (*TLR1, TLR6, TLR7, TLR12*), cytosolic DNA sensing, NOD-like, RIG-I-like pathways contained up-regulated genes showing that the extra and intracellular forms of the pathogen could be recognized and could induce an inflammatory response. Among the cytokine signaling pathways, IFN-related signaling pathways and IFN-induced gene expressions were particularly noticeable, including up-regulated genes *IFNG, STAT1, STAT2, IRF7, IRF9*, and IFN-induced genes such as several histocompatibility complex genes, IFN-inducible GTPases and tryptophan catabolism genes *IDO1* and *KYNU*.

### Antimicrobial Genes Induced by *C. muridarum* Infection

A prominent identified Gene Ontology molecular functional category was “GTP binding.” Most of the GTPases in this category are involved in defense responses against intracellular pathogens (Figure 2B). Essentially all four classes of murine IFN-inducible GTPases were found to be up-regulated including various guanylate-binding proteins (*GBP2-9, GBP11*), the myxovirus resistance protein-1 (*MX1*), immunity-related GTPases *IRGM1* (*LRG47*), *IRGM2* (*GTPI*), *IRGA6* (*IIGP*), *IRGM3* (*IGTP*), *IRGB6* (*TGTP1-2*), *IRGB10* (*Gm12250*), and a very large IFN-inducible GTPase *GVIN1*. It is worth to note, that three predicted genes with a sequence homology to *IIGP* (*Gm4841, Gm4951, F830016B08Rik*) and three with a sequence homology to *TGTP* (*Gm12185, Gm5431, 9930111J21Rik1*) were found to be up-regulated or highly up-regulated in the infected lungs (Figure 2B). Interestingly, *Gm12185, Gm5431*, and *9930111J21Rik* are localized on chromosome 11, close to the region where potential defense genes against *C. trachomatis* were found by QTL mapping (Bernstein-Hanley et al., 2006a,b). Besides the IFN-inducible GTPases genes, other genes such as *CXCL9, iNOS*, and *IDO1* with known antichlamydial activity against human and murine *Chlamydia* strains were highly up-regulated (127 fold, 14 fold, and 24.7 fold, respectively). Additional genes with known antimicrobial activity, but unknown antichlamydial activity were found to be up-regulated including *CXCL11* (345 fold), *IRG1* (252.7 fold), lipocalin-2 (11 fold), mucin-5 (8.7 fold) and solute carrier family 11 (5.5 fold).

### qPCR Validation of *IDO1* and *IDO2* RNA-Seq Data

**Figure 3:**
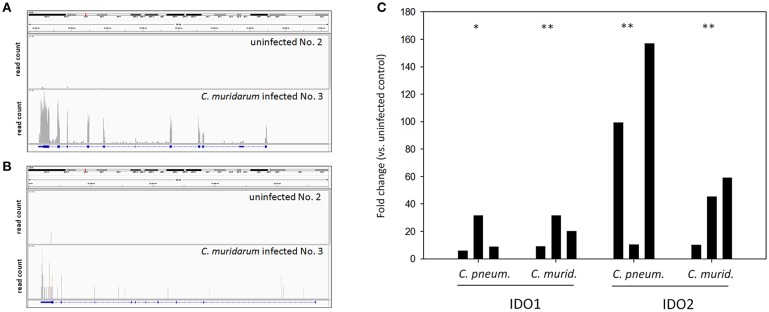
*IDO1* and *IDO2* mRNA expression measurements by RNA-Seq and qPCR. Integrative Genomics Viewer (Thorvaldsdóttir et al., 2013) images showing the coverage of *IDO1* **(A)** and *IDO2* **(B)** genes by sequencing reads in a representative *C. muridarum* infected and uninfected control samples. *IDO1* and *IDO2* gene expression inductions measured by qPCR in *C. muridarum* and *C. pneumoniae* infected lung tissues (*n* = 3) **(C)**. *IDO1* and *IDO2* gene expression fold changes in each infected mice (*n* = 3) were calculated by comparing the average *IDO1* and *IDO2* expressions in the uninfected controls (*n* = 3). *IDO1* and *IDO2* gene expressions were normalized by the beta-actin gene expressions. ΔCt values (Ct_*IDO*_-Ct_*actin*_) of the infected and uninfected samples was compared by Student's *t*-test. **P < 0.01 *P < 0.05.

The fact that the *IDO1* gene was highly upregulated (24.76 fold) in the infected lungs was an unexpected finding since the *IDO1* was found to be non-inducible by *C. trachomatis* or *C. muridarum* infection *in vitro* in murine epithelial and other cells (Nelson et al., 2005; Roshick et al., 2006; Burian et al., 2010). *IDO1* was detected as a significantly changed gene (*P* = 0.037) between the *C. muridarum* infected and uninfected samples, while the *IDO2* was detected non-significant. However, as Figures 3A,B shows, both *IDO1* and *IDO2* genes had more sequence reads in the infected samples than the uninfected ones. Yet, in the case of *IDO2*, the read numbers were not high enough to be detected as a significantly changed gene. We used qPCR as an independent method to validate RNA-Seq data for *IDO1* and *IDO2*. Also, in order to test whether the *IDO1-2* induction is unique to the murine *Chlamydia* strain, we measured the *IDO1-2* gene expressions in *C. pneumoniae* infected lung samples. qPCR data supported the RNA-Seq data in the case of *C. muridarum* infection with a 20.38 ± 11.3 fold and 38.2 ± 25.2 fold of upregulation of *IDO1* and *IDO2*, respectively. qPCR also showed a similar extent of up-regulation 15.5 ± 14.1 fold and 88.9 ± 73.9 fold for *IDO1* and *IDO2*, respectively in the *C. pneumoniae* infected lung tissues (Figure 3C).

### IDO1-2 Protein Expression in *C. muridarum* and *C. pneumoniae* BALB/c Infected Mouse Lung Tissues

**Figure 4:**
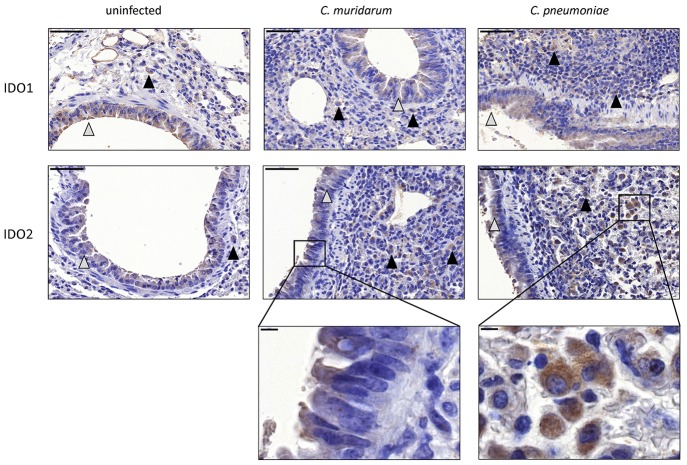
Detection of IDO1-2 protein expressions in *Chlamydia* infected and uninfected BALB/c mouse lungs. IDO1 protein and IDO2 protein expressions detected by immunohistochemistry in *C. muridarum* infected, *C. pneumoniae* infected (7 days post infection) and uninfected control lung tissues. The IDO positive epithelial cells are shown by gray triangles, the IDO positive macrophages are shown by black triangles. Bars: 50 μm. The characteristic IDO stainings of epithelial cells and macrophages are shown in brackets. Bars: 5 μm.

To prove that the observed *IDO1* and *IDO2* mRNA changes translated to protein expression changes, and to localize the cell type(s) that express these proteins, we performed an immunohistochemistry of *C. muridarum* and *C. pneumoniae* infected lung sections and uninfected controls. Moderate IDO1-2 positivity could be detected in the cytoplasm of bronchial and occasionally alveolar epithelial cells and moderate/strong positivity was detected frequently in macrophages in the *C. pneumoniae* and *C. muridarum* infected mouse lung tissues (Figure 4). *C. pneumoniae* and *C. muridarum* infections lead to similar IDO1-2 positivity in these cells. The control, uninfected lung tissues also contained IDO1-2 positive bronchial epithelial cells, and a small number of IDO1-2 positive macrophages.

### IDO 1-2 Activity in *C. muridarum* and *C. pneumoniae* Infected BALB/c Mouse Lung Tissues

**Figure 5:**
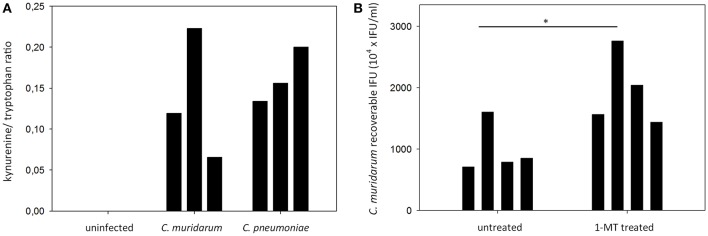
Measurement of tryptophan degradation in the *Chlamydia* infected BALB/c lung tissues and the effect of IDO1-2 inhibition on *C. muridarum* growth. **(A)** *C. muridarum* infected, *C. pneumoniae* infected and uninfected lung tissues (*n* = 3) were processed as described in the Materials and Methods for kynurenine and tryptophan concentration measurements at 7 days post infection. Tryptophan degradation was described by measuring the kynurenine/tryptophan ratio. Kynurenine/tryptophan ratios of each of the lungs analyzed are shown. Kynurenine concentrations of the uninfected samples were below the limit of detection. **(B)** untreated (*n* = 4) and 1-MT treated (*n* = 4) BALB/c mice were infected with *C. muridarum* and the recoverable IFUs were measured at 7 days post infection. Recoverable IFUs from each of the analyzed lungs are shown. Recoverable IFUs from untreated and 1-MT treated samples were compared by Student's *t*-test. *P < 0.05.

To determine whether the expressed IDO1 and IDO2 proteins were functional, we performed a HPLC analysis of the infected and control lung tissues of mice included in the gene expression and immunohistochemistry measurements. We measured IDO1-2 activity by measuring the total tryptophan level, and the level of the tryptophan degradation metabolite kynurenine. The applied HPLC method could not detect kynurenine in the uninfected lungs, while the *C. muridarum* and *C. pneumoniae* infected lungs contained 369.6 ± 199.8 nM and 508.7 ± 176.6 nM. Since we could not control the cell numbers in the infected and control tissues, we normalized the samples by using the kynurenine/tryptophan ratios as described previously (Veres et al., 2015). The kynurenine/tryptophan ratios ranged from 0.12 to 0.22 in the *C. muridarum* infected samples, 0.13–0.20 in the *C. pneumoniae* infected samples and it was 0 in the control samples (Figure 5A). To assess the impact of IDO activity on *C. muridarum* growth we inhibited IDO1-2 by 1-MT treatment starting from seven days before infection to seven days post infection (Figure 5B). 1-MT treatment lead to a moderate but significant, 1.98 fold increase in *C. muridarum* recoverable IFU at 7 days post infection.

### IDO 1-2 mRNA Expression and Activity in *C. muridarum* Infected C57BL/6 Mouse Lung Tissues

**Figure 6:**
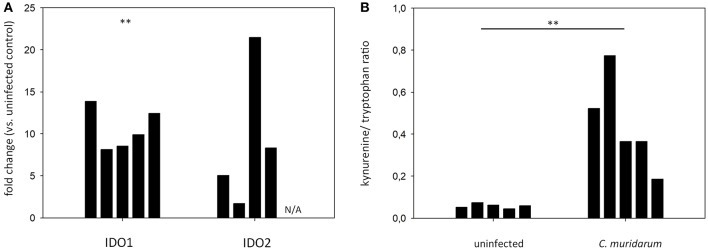
qPCR measurement of *IDO1* and *IDO2* gene expressions in the *C. muridarum* infected C57BL/6 lung tissues **(A)**. *IDO1* and *IDO2* gene expression fold changes in each infected mice (*n* = 5) were calculated by comparing the average *IDO1* and *IDO2* expressions in the uninfected controls (*n* = 5). *IDO1* and *IDO2* gene expressions were normalized by the beta-actin gene expressions. ΔCt values (Ct_*IDO*_-Ct_*actin*_) of the infected and uninfected samples was compared by Student's *t*-test. **P < 0.01. N/A: IDO2 mRNA expression could not be detected. **(B)** Measurement of tryptophan degradation in the *C. muridarum* infected C57BL/6 lung tissues. *C. muridarum* infected and uninfected lung tissues (*n* = 5) were processed as described previously at 7 days post infection. Kynurenine/tryptophan concentration ratios of each of the lungs analyzed are shown. Kynurenine/tryptophan concentration ratios of *C. muridarum* infected and uninfected samples were compared by Student's *t*-test. **P < 0.01.

To explore whether the *Chlamydia*-induced IDO1-2 activity could be observed in another mouse strain, we performed qPCR and HPLC analyses of *C. muridarum* infected and control lung tissues of C57BL/6 mice. qPCR data showed a significant increase of IDO1 mRNA level in the *C. muridarum* infected lungs (fold of up-regulation range: 8.14–13.88), and while the IDO2 mRNA up-regulation was not significant, an up-regulation tendency could be observed (fold of up-regulation range: 1.71–21.49) (Figure 6A). HPLC analysis of tryptophan and kynurenine contents showed that uninfected C57BL/6 mice lungs contained a small amount of kynurenine (0.045–0.075 kynurenine/tryptophan concentration ratios), and the *C. muridarum* infection significantly increased the IDO activity (0.185–0.773 kynurenine/tryptophan concentration ratios) (Figure 6B).

## Discussion

RNA sequencing analysis of the *C. muridarum* infected lungs revealed that the expression of a wide variety of host genes were altered, and several up-regulated genes could contribute in the *Chlamydia*-induced inflammation and antichlamydial defense of the murine host. Genes related to both the innate and adaptive immunity were found to be induced in the *C. muridarum* infected lung tissue. The major functional “themes” were related to cytokine/chemokine expression, chemotaxis, signal transduction, antigen presentation, cell division and innate antimicrobial defense. According to the cellular theory of chlamydial pathogenesis, non-immune cells trigger the inflammation by secreting proinflammatory cytokines and chemokines (Stephens, 2003). While the cellular source of the cytokines/chemokines cannot be identified by a tissue-level gene expression analysis, *in toto* the strong gene expression imprint of chemotaxis induction and cellular influx could be identified in the *C. muridarum* infected mouse lungs. The autocrine-paracrine effects of the secreted cytokines and the cell-to-cell interactions between resident and novel cells could result in a milieu that induced a complex gene expression including the induction of certain antichlamydial genes.

Several members of the IFN-inducible GTPase family were found to be highly induced. Although we did not measure the gene expression changes induced by the *C. pneumoniae*, previous studies showed that both the murine and human *Chlamydia* strains were able to induce IFN-inducible GTPases (Nelson et al., 2005; Burian et al., 2010). GTPases *IRGM1* (*LRG47*), IRGM3 (*IGTP*), *IRGA6* (*IIGP*), and *IRGB10* were found to be upregulated or highly-upregulated, and have been shown to be involved in the clearance of the human *C. trachomatis* strain (Nelson et al., 2005; Bernstein-Hanley et al., 2006b; Coers et al., 2008), but likely not effective against the murine *Chlamydia* (Nelson et al., 2005; Coers et al., 2008). Novel GTPase genes were also found to be highly up-regulated after *C. muridarum* infection, and could be involved in the antichlamydial defense. Three of these genes (*Gm12185, Gm5431, 9930111J21Rik1*) show a sequence similarity to *IRGB10*, a known murine defense gene against *C. trachomatis* (Bernstein-Hanley et al., 2006b). Interestingly, Coers et al. showed that *C. muridarum* was capable of evading the antichlamydial effect of IRGB10 (Coers et al., 2008). Altogether, the differential sensitivity to the IFN-inducible GTPases could explain the fact that despite using 400 fold more *C. pneumoniae* IFU than *C. muridarum*, we recovered comparable IFUs from the infected lungs. The other known antichlamydial gene *iNOS* was also up-regulated (14 fold). iNOS induction has been shown to be an important mechanism in the later phase elimination of *C. muridarum* infection from the mouse genital tract (Johnson et al., 2012) and also in RAW 264.7 murine macrophages (Rajaram and Nelson, 2015). *IRG1*, another IFN-inducible gene (Tallam et al., 2016) (Naujoks et al., 2016) was found to be highly up-regulated (252.7 fold) after *C. muridarum* infection. IRG1 was shown to be expressed in macrophages (Hall et al., 2013; Naujoks et al., 2016), associated with *Legionella pneumophila* vacuoles in macrophages and possessed antimicrobial activity by increasing reactive oxygen species production (Hall et al., 2013) and by the direct bactericidal effect of itaconic acid production (Naujoks et al., 2016). A CXC chemokine, *MIG* was also highly up-regulated (127 fold) after *C. muridarum* infection. We showed previously that MIG had a concentration-dependent direct toxicity to the elementary bodies of *C. muridarum, C. trachomatis* (Burian et al., 2010) and *C. pneumoniae* (Balogh et al., 2011).

RNA sequencing and qPCR revealed that the *IDO1* and *IDO2* genes were also highly induced in the infected lungs. To identify the source of IDO activity, we performed IDO1-2 IHC in infected and control lung tissues. We found that lung bronchial epithelial cells had a moderate level of IDO1-2 positivity both in the control and infected tissues, indicating a lower-level, steady-state expression. A higher level of IDO1-2 positivity was detected in leukocytes, prominently in macrophages, in both the uninfected and infected tissues, but the number of positive cells was higher in the *Chlamydia*-infected tissues. The higher number of IDO1-2 positive macrophages might be a result of *in situ* IDO1-2 induction and/or the influx of already IDO1-2 positive monocytes into the inflamed tissue. It is also possible that the IDO1-2 positive macrophages were activated locally resulting in a higher IDO1-2 activity. Previous *in vivo* studies showed a tissue level IDO induction in various murine tissues infected by *Escherichia coli* (Loughman and Hunstad, 2012)*, Plasmodium berghei* (Sanni et al., 1998), and *Toxoplasma gondii* (Fujigaki et al., 2002). Interestingly, *C. pneumoniae* induced lung IDO1 expression was also shown in RAG^−/−^ mice, but not in the wild type C57BL/6 controls (Rottenberg et al., 2000). Cellular level IDO induction *in vivo* was detected in murine intestinal epithelial cells after *Eimeria falciformis* infection (Schmid et al., 2012), in lung epithelial cells after influenza A virus infection (Huang et al., 2013) and in lung epithelial cells, endothelial cells and macrophages/dendritic cells after *Mycobacterium tuberculosis* infection (Desvignes and Ernst, 2009). Knockout studies showed that—similarly to humans—one of the major inducers of IDO expression is IFNG and IFNA/B in murine tissues *in vivo* (Loughman and Hunstad, 2012; Huang et al., 2013). According to our gene expression data one of the prominent networks induced by *C. muridarum* infection was the IFN signaling pathway, therefore the IFN impact of *IDO1-2* gene induction was clearly present in the *Chlamydia*-infected tissues. HPLC detection of the tryptophan degradation product kynurenine in BALB/c lungs demonstrated that *i*, in the uninfected murine lung tissues IDO activity was not detectable, hence the low level IDO1-2 protein positivity detected in uninfected epithelial cells and macrophages did not yield significant tryptophan catabolism *ii*, IDO1-2 enzymes were induced and functionally active in both the murine and human *Chlamydia* infected lung tissues. A quantitative IHC was not performed, but the observation of similar level of IDO1-2 IHC positivity in epithelial cells before and after infection indicates that the IDO1-2 were not induced and IDO1-2 activity might not be involved in the elimination of *Chlamydia* from the murine lung epithelial cells. Further quantitative studies needed to clarify the exact role of murine epithelial IDO expression. The fact that the C57BL/6 mouse lungs also showed *Chlamydia* infection induced IDO activity supports that the observed IDO induction is not a mouse strain-specific response.

The role of murine IDO in the clearance of *Chlamydia in vivo* is not well-defined. IDO1 knockout mice cleared *C. muridarum* from the urogenital tract with similar—but not identical—kinetics to the wild types (Nelson et al., 2005), but it should be noted that IDO2 gene was intact in these animals (Mellor et al., 2003; Blumenthal et al., 2012). To clarify the antimicrobial role of IDO1-2 activity we treated BALB/c mice with 1-MT, a previously described inhibitor of IDO1 (Cady and Sono, 1991) and IDO2 (Metz et al., 2007). IDO inhibition showed that there was a moderate but significant, ~2 fold increase in *C. muridarum* recoverable IFU in 1-MT treated mice indicating that IDO activity influenced the *C. muridarum* replication *in vivo*.

Both the *C. muridarum* and *C. pneumoniae* infections clearly lead to the presence of IDO1-2 positive macrophages, and this leukocyte influx/activation was a major factor in the observed increase in IDO1-2 activity. Monocytes have been implicated in the spread of *C. pneumoniae* from the primary site of infections (Beagley et al., 2009), but human monocytes were able to suppress *C. trachomatis* growth via IDO-dependent (Carlin and Weller, 1995) and IDO-independent mechanisms (Koehler et al., 1997; Nettelnbreker et al., 1998; Marangoni et al., 2014). Whether the IDO activity of murine macrophages contributes to the control of *C. muridarum* and *C. trachomatis* growth, needs further investigation.

There are limitations of our study that need further investigations. We could not detect IDO 1-2 inducibility in epithelial cells, but the applied IHC was not a quantitative method. Since epithelial cells are the sites of chlamydial replication, the isolation of lung epithelial cells and the measurement of their steady state and infection-induced IDO activity are critical points and a goal we are currently pursuing. The other obvious targets to assess IDO activity are the isolated epithelial cells of the uninfected and infected murine urogenital tract. The role of IDO2 is not defined. The lung IDO2 mRNA was clearly induced by *Chlamydia* infection, but its RNA-seq read numbers were significantly lower than IDO1 reads. In order to assess the potential role of IDO2, the IDO2 protein concentrations in isolated lung epithelial cells and macrophages has to be measured and compared to IDO1. Also, chemical inhibition of IDO showed a significant, albeit limited phenotypic effect. This could be due to the limited defensive role of IDO 1-2 or the incomplete inhibition of the enzymes. Further studies with optimized IDO inhibition protocol and more time points post infection are needed.

*Chlamydia* can avoid intracellular defense responses by using metabolic shunt (Aiyar et al., 2014), inactivating cellular effector proteins such as IFN-inducible GTPases (Nelson et al., 2005; Coers et al., 2008), or avoiding the induction of the intracellular effectors (Marangoni et al., 2014). Our *in vivo* study showed that –at the tissue level- various antibacterial mechanisms are switched on and IDO1-2 could be part of this effector repertoire.

## Data Availability

The raw data analyzed in this study can be found in the Gene Expression Omnibus (GEO) database (GSE124007). The lists of differentially expressed genes are included in the Supplementary Table 1.

## Ethics Statement

The experiments were approved by the Animal Welfare Committee of the University of Szeged and conform to the Directive 2010/63/EU of the European Parliament.

## Author Contributions

DV designed the experiments, analyzed the RNA-seq data and prepared the manuscript. KB, TR, and VE were involved in designing the experiments, performed the animal infections, tissue extraction, *Chlamydia* growth monitoring, histology, and manuscript preparation. DK, DP, and AB were involved in manuscript preparation, identification of novel GTPases and phylogeny of GTPases. LT performed the histology and IDO1-2 immunohistochemistry. LV and GV performed the kynurenine and tryptophan HPLC measurements. LV critically reviewed the manuscript. SP performed the RNA sequencing, identification of differentially expressed genes, collection and comparison of IDO1 and IDO2 raw data. FS performed the *IDO1-2* primer designs, *IDO1-2* qPCRs and was involved in higher level RNA-seq analysis.

### Conflict of Interest Statement

The authors declare that the research was conducted in the absence of any commercial or financial relationships that could be construed as a potential conflict of interest.

## Supplementary Material

Supplementary Table 1 Differentially expressed genes in *C. muridarum* infected vs. uninfected BALB/c lung tissues.
